# Small airways dysfunction in patients with systemic sclerosis and interstitial lung disease

**DOI:** 10.3389/fmed.2022.1016898

**Published:** 2022-11-14

**Authors:** Panagiotis K. Panagopoulos, Andreas V. Goules, Vasiliki E. Georgakopoulou, Anastasios Kallianos, Eirini Chatzinikita, Vasileios C. Pezoulas, Katerina Malagari, Dimitrios I. Fotiadis, Panayiotis Vlachoyiannopoulos, Theodoros Vassilakopoulos, Athanasios G. Tzioufas

**Affiliations:** ^1^Department of Pathophysiology, School of Medicine, National and Kapodistrian University of Athens, Athens, Greece; ^2^Research Institute of Systemic Autoimmune Diseases, Athens, Greece; ^3^Department of Physiology, School of Medicine, National and Kapodistrian University of Athens, Athens, Greece; ^4^Unit of Medical Technology and Intelligent Information Systems, University of Ioannina, Ioannina, Greece; ^5^2nd Department of Radiology, “Attikon” Hospital, University of Athens, Athens, Greece

**Keywords:** systemic sclerosis, interstitial lung disease, small airways dysfunction, pulmonary function tests, phase III slope_N2SBW_, R5–R20

## Abstract

**Background:**

A number of studies report small airways involvement in patients with systemic sclerosis (SSc). Furthermore, small airways dysfunction is increasingly recognized in patients with interstitial lung disease (ILD) of idiopathic or autoimmune etiology. The objectives of this study were to evaluate small airways function in SSc patients with ILD and explore the effect of treatment on small airways function by using conventional and contemporary pulmonary function tests (PFTs).

**Methods:**

This single-center, prospective, observational study included a total of 35 SSc patients, with and without ILD based on HRCT scan, evaluated by a special radiologist blindly. Clinical data were collected from all patients who were also assessed for HRCT findings of small airways disease. Small airways function was assessed by classic spirometry, measurement of diffusing capacity for carbon monoxide, body plethysmography, single breath nitrogen washout (N_2_SBW) and impulse oscillometry (IOS). The prevalence of small airways dysfunction according to R5–R20, phase III slope_N2SBW_ and CV/VC methodologies was calculated in the total SSc population. Pulmonary function tests were compared between: (a) SSc-ILD and non-ILD patients and (b) two time points (baseline and follow up visit) in a subset of SSc-ILD patients who received treatment for ILD and were re-evaluated at a follow up visit after 12 months.

**Results:**

Phase III slope_N2SBW_ and R5–R20 showed the highest diagnostic performance for detecting small airways dysfunction among SSc patients (61 and 37.5%, respectively). Twenty three SSc patients were found with ILD and 14 of them had a 12-month follow up visit. SSc-ILD patients compared to those without ILD exhibited increased phase III slope_N2SBW_ ≥120% (*p* = 0.04), R5–R20 ≥0.07 kPa/L/s (*p* = 0.025), airway resistance (Raw) (*p* = 0.011), and special airway resistance (sRaw) (*p* = 0.02), and decreased specific airway conductance (sGaw) (*p* = 0.022), suggesting impaired small airways function in the SSc-ILD group. Radiographic features of SAD on HRCT were observed in 22% of SSc-ILD patients and in none of SSc-non-ILD patients. Comparison of PFTs between baseline and follow-up visit after 12 months in the 14 SSc-ILD treated patients, showed improvement of phase III slope_N2SBW_ (*p* = 0.034), R5–R20 (*p* = 0.035) and Raw (*p* = 0.044) but not sRaw and sGaw parameters.

**Conclusion:**

Phase III slope_N2SBW_ and R5–R20 may reveal small airways dysfunction in SSc associated ILD before structural damage and may be partially improved in a subset of patients receiving treatment for ILD.

## Introduction

Systemic sclerosis (SSc) is a systemic autoimmune disorder which is pathogenetically characterized by microvascular injury, dysregulation of innate and adaptive immunity and tissue fibrosis in different organs (1). Microvascular involvement and endothelial cell injury are early pathogenetic events in SSc, leading to Raynaud's phenomenon and characteristic capillaroscopic findings (2, 3). Immunologic derangements include aberrations of T and B cell subset homeostasis, infiltration of affected tissues by T and B cells, plasma cells and macrophages, production of autoantibodies with pathogenetic role and release of profibrotic cytokines by B cells (4). Although skin fibrosis is usually the most prominent presenting feature of the disease, pulmonary, gastrointestinal, renal and heart involvement determine often the final clinical outcome (1). Pulmonary manifestations of SSc include interstitial lung disease (ILD), pulmonary arterial hypertension (PAH) and pleural disease, with ILD and PAH being the leading causes of morbidity and mortality among these patients (5).

Interstitial lung disease affects about 40–60% of SSc patients and is histopathologically characterized by different degrees of parenchymal inflammatory infiltration and fibrosis. Diagnosis of ILD in these patients is primarily made by high resolution computed tomography (HRCT) of the lungs. The most frequent radiographic pattern of ILD in this disease is fibrotic non-specific interstitial pneumonia (NSIP), followed by cellular NSIP and usual interstitial pneumonia (UIP) (6). Interestingly, the imaging pattern may predict adverse outcomes in cases of transbronchial cryobiopsy for diagnostic purposes or disease progression after COVID-19 pneumonia in SSc patients (7, 8). Pulmonary function tests (PFTs) are a useful, non-invasive tool, with no radiation exposure, for the screening, diagnosis and monitoring of ILD in SSc (SSc-ILD). They are characterized by a restrictive pattern with decreased forced vital capacity (FVC), as well as by defective gas transfer in even early stages, as manifested by diffusing capacity for carbon monoxide (DLCO) (9).

Although the airways are not classically involved in SSc, a number of studies report small airways involvement in SSc patients with or without ILD (10–16). Furthermore, small airways disease (SAD) is increasingly recognized in patients with ILD of idiopathic or autoimmune etiology (17–22). Small airways dysfunction may be evaluated by imaging and by pulmonary function tests. HRCT findings in SAD include mosaic attenuation and hyperlucent areas implying air trapping on expiratory CT scan (23). Classic spirometry cannot reliably reflect airflow through peripheral airways (24, 25). Yet pulmonary function tests are available to assess small airway function and have been recently widely introduced in clinical practice with the advent of modern technology. Nitrogen single-breath washout technique (N_2_SBW) assesses ventilation homogeneity and small airways dysfunction. Change in the concentration of nitrogen between 25% and 75% of the exhaled volume (phase III slope_N2SBW_) and the closing volume/vital capacity ratio (CV/VC) reflect peripheral airways function, with higher values implying small airways dysfunction (16). Furthermore, impulse oscillometry (IOS) is a variant of forced oscillation technique that has been used for the study of small airways. Resistance at low frequencies, such as 5Hz (R5), reflects total airways resistance, whereas resistance at higher frequencies, such as 20Hz (R20), reflects larger airways resistance. Therefore, the difference between resistance at 5Hz and 20Hz (R5–R20) is an estimate of small airways resistance. Moreover, reactance at 5 Hz (X5) and resonance frequency (Fres) are other IOS parameters associated with small airway function (26). Interestingly, airways resistance (Raw) and specific airways resistance (sRaw), measured by body plethysmography, are increased in airflow obstruction, while specific airways conductance (sGaw) (which is the inverse of sRaw) is decreased. Raw and sRaw reflect central and peripheral airways obstruction, whereas sGaw seems to be a more sensitive measure of small airways dysfunction (24, 25). Respiratory resistance may also be measured by the interrupter technique (R_int_) (27).

The objectives of this prospective study were: i. to evaluate lung and small airways function in SSc patients with ILD by employing distinct pulmonary function tests simultaneously for the first time in the literature, assuming that they are more sensitive than HRCT in detecting small airway dysfunction, and ii. to assess the effect of ILD related treatment upon small airways function in SSc-ILD patients.

## Patients and methods

### Patients' cohort

This single-center, prospective, observational study included consecutive SSc patients fulfilling the 2013 ACR/EULAR classification criteria for SSc (28), who visited the outpatient rheumatology clinic of the Department of Pathophysiology between September 2020 and May 2022 and underwent HRCT scans either as part of standard of care or due to respiratory complaints. Clinical, laboratory, immunologic and imaging data were collected from all SSc patients and all patients underwent conventional and contemporary pulmonary tests at the time of recruitment. Immunological work-up included indirect immunofluorecence for antinuclear antibodies (ANA) and their staining pattern (including anti-centromere antibodies, ACA) on commercially available Hep-2 cells and ethanol- fixed neutrophils using the NOVA Lite HEp-2 ANA kit (Inova Diagnostics Inc, San Diego, CA, USA), according to manufacturer's instructions. Immunoblotting for extractable nuclear antigen antibodies (ENA) was performed, including anti-Scl-70/anti-topoisomerase I (ATA), using the Euroline Anti-ENA ProfilePlus1 (IgG) kit (Euroimmun, Lübeck, Germany).

HRCT scans were evaluated blindly by a special radiologist, according to Fleischner Society definitions (29), for the presence of ILD, the imaging pattern (fibrotic NSIP, cellular NSIP, UIP), and the presence of radiographic features of SAD (mosaic attenuation, hyperlucent areas implying air trapping on expiratory CT scan). The extent of ILD on HRCT was classified as limited (<20%) or extensive disease (≥20%) according to Goh et al. (30). Pulmonary and small airways function tests included classic spirometry, measurement of DLCO, body plethysmography, single breath nitrogen washout, impulse oscillometry and measurement of R_int_. We utilized three distinct tests to evaluate and compare prevalence of small airways dysfunction among SSc patients: phase III slope_N2SBW_ (expressed as a percentage of the predicted value) ≥120% (16), CV/VC (% predicted) ≥120% (16) and R5–R20 ≥ 0.07 kPa/L/s (11). A more detailed description of all pulmonary tests is provided in the Supplementary material, “Patients and Methods” section.

Treatment in all SSc patients was administered according to physician's judgment as part of standard of care. Some SSc-ILD patients, receiving different therapeutic regimens, performed a second follow-up visit after 1 year, and underwent the same conventional and contemporary pulmonary function tests.

The study was approved by the Ethics Committee of the School of Medicine, National and Kapodistrian University of Athens. The patients were informed about the nature of the study and the investigations performed and they gave written consent to their participation in the study.

### Statistical analysis

Statistical analysis for categorical data was performed by Fisher exact test when cell counts <5 patients or χ2 square test with Yates correction accordingly and numerical data with Man-Whitney test or *t*-test after applying the Shapiro-Wilk normality test. The level of statistical significance was set at 0.05. Statistical analysis was performed in Python 3.6 and GraphPad 7.0a. In order to handle the multiple comparison testing, the original *p*-values were also adjusted with the Benjamini-Hochberg (B-H) procedure using 0.1 as the false discovery rate (31).

## Results

### Characteristics of SSc patients' cohort

The study included 35 SSc patients, 23 with and 12 without ILD based on HRCT imaging classification (SSc-ILD and SSc-non-ILD, respectively) while 14 of 23 SSc-ILD patients had a second follow up visit after 12 months. The mean ± SD age of the 35 SSc patients was 61.1 ± 10.7 years and the majority were females (91%). Eighteen (*n* = 18) patients (51%) had smoking history but none suffered from COPD, asthma or bronchiectasis. The median disease duration was 6.3 years (range: 0.5–33 years), while median treatment duration was 2.5 years (range: 0–13.5 years). Thirty four % of SSc patients had the diffuse cutaneous form of the disease. Twenty one (*n* = 21) patients (60%) presented exertional dyspnea and 4 (11%) had dry cough. All SSc had positive ANA, 71% had positive ATA, whereas ACA pattern was present in 17% of patients. Nineteen (*n* = 19) patients (54%) had received mycophenolate mofetil, 16 (46%) cyclophosphamide, 8 (23%) rituximab, 6 (17%) methotrexate and 3 (9%) nintedanib (Supplementary Table 1).

Cellular NSIP pattern on HRCT was observed in 48% of SSc-ILD patients, whereas 52% had fibrotic NSIP imaging pattern. The most frequent finding on HRCT in SSc-ILD patients was ground-glass opacities (83%), followed by fine reticulation (65%). Coarse reticulation was observed in 13% and traction bronchiectasis in 26% of SSc-ILD patients. Thirty-nine percent of SSc-ILD patients had extensive disease on HRCT (extent of ILD >20%). It should be noted that HRCT findings suggestive of small airways disease (mosaic attenuation or air trapping) were observed in 22% of SSc-ILD patients and in none of the SSc-non-ILD patients.

### Comparison of different small airways pulmonary function tests among SSc patients

In the total cohort of 35 SSc patients, the prevalence of small airways dysfunction based on 3 distinct methods was estimated (Table 1). The prevalence of small airways dysfunction based on R5–R20, phase III slope_N2SBW_ (expressed as a percentage of the predicted value) and predicted CV/VC was 37.5, 61, and 12%, respectively (Table 2). Only 4 of 19 (21.5%) SSc patients had small airway dysfunction assessed with at least 2 different tests which were R5–R20 and phase III slope_N2SBW_; these patients presented worse conventional pulmonary tests including DLCO and had more often ILD findings in HRCT (data not shown). Seven of 15 (45%) SSc patients that had normal R5–R20 values had abnormal phase III slope_N2SBW_ tests and 3 of 9 (33%) SSc patients that had normal phase III slope_N2SBW_ had abnormal R5–R20 test values, implying that the 2 methods should be used complementary, although phase III slope_N2SBW_ had better overall performance (Table 2). Since, phase III slope_N2SBW_ exhibited the highest diagnostic yield for small airways dysfunction, we proceeded by comparing SSc patients with normal and abnormal values; the latter group had statistically significant higher frequency of ILD determined by HRCT, worse conventional pulmonary function tests, yet comparable HRCT findings for small airways disease (Supplementary Tables 2, 3). The predicted DLCO was comparable between R5–R20 and phase III slope_N2SBW_ positive patients (62 vs. 69%) and the predicted FEF_25%−75%_ was less impaired in phase III slope N2 positive patients (62 vs. 74%).

**Table 1:** R5-R20, phase III slope_N2SBW_ and CV/VC tests in SSc patients.

**Patient ID#**	**R5–R20 ≥0.07 kPa/L/s**	**Phase III slope_N2SBW_ (% pred) ≥120%**	**CV/VC (% pred) ≥120%**	**HRCT findings suggestive of small airways disease**
1	N/A	1	0	0
2	0	1	0	0
3	1	1	0	0
4	1	0	0	0
5	1	N/A	N/A	0
6	N/A	1	1	0
7	0	1	0	0
8	N/A	N/A	N/A	0
9	N/A	0	0	1
10	N/A	N/A	0	1
11	0	1	0	1
12	N/A	N/A	N/A	0
13	N/A	N/A	N/A	1
14	1	1	0	0
15	0	1	0	0
16	0	N/A	N/A	0
17	N/A	N/A	N/A	0
18	N/A	N/A	N/A	0
19	0	1	0	0
20	1	1	0	1
21	0	0	0	0
22	1	N/A	1	0
23	1	0	0	0
24	0	0	1	0
25	0	1	0	0
26	0	1	0	0
27	0	0	0	0
28	0	N/A	N/A	0
29	1	1	0	0
30	0	N/A	N/A	0
31	N/A	1	0	0
32	1	0	0	0
33	0	0	0	0
34	0	0	0	0
35	N/A	N/A	N/A	0

N/A, non-available; 0, negative test; 1, positive test.

**Table 2:** Pulmonary function parameters of SSc patients with SAD diagnosed with the use of R5-R20, phase III slope_N2SBW_ and CV/VC.

	**R5–R20** **≥0.07 kPa/L/s**	**phase III slope_N2SBW_** **(% pred) ≥120%**	**CV/VC** **(% pred) ≥120%**
Prevalence of small airways dysfunction	37.5% (9/24)	60.87% (14/23)	12% (3/25)
FVC (% pred)	98.7 ± 16.4	91.9 ± 16.6	81 (79–117)
FEV1/FVC (%)	76.7 ± 5.8	80.2 ± 5.1	70.4 (70.3–79.8)
FEF_25−75_ (% pred)	62.2 ± 19.9	74.3 ± 26.7	41 (39–113)
PEF (% pred)	87.8 ± 17.4	103.9 ± 16	65 (63–101)
DLCO (% pred)	68.9 ± 26.7	62.1 ± 20	79 (51–120)
R5–R20 ≥0.07 kPa/L/s	Not applicable	4 (29%)	1 (50%)
phase III slope_N2SBW_ (% pred) ≥120%	4 (57%)	Not applicable	1 (50%)
CV/VC (% pred) ≥120%	1 (13%)	1 (7%)	Not applicable
sRaw (cmH2O*s)	8.36 ± 2.33	5.86 (3.4–9.84)	5.17 (1.5–13.07)
Raw (cmH2O*s/L)	3.3 (1.97–4.97)	2.7 (1.83–4.97)	3.3 (0.55–2.12)
sGaw (1/cmH2O/s)	0.12 (0.08–0.23)	0.18 (0.06–0.31)	0.2 (0.08–0.72)
Gaw (L/cmH2O/s)	0.31 (0.2–0.51)	0.38 (0.06–0.57)	0.48 (0.31–1.82)
R_int_ (% pred)	125.5 (81–164)	93.9 ± 25.3	86 (69–128)

### Comparison of SSc patients with and without radiographically detected ILD

Gender did not differ significantly between SSc-ILD and SSc-non-ILD patients (91% females vs. 92% females, respectively, *p* = 0.098), while SSc-ILD patients were older than SSc-non-ILD patients (mean: 62.3 vs. 58.7 years old, *p* = 0.039). SSc-ILD patients had significantly shorter disease duration than SSc-non-ILD patients (median: 5 vs. 12.1 years, *p* = 0.017). Smoking history did not differ significantly between the two groups (48% of SSc-ILD patients vs. 58% of SSc-non-ILD patients had a history of ever smoking, *p* = 0.091). Ninety one (91) % of SSc-ILD patients had positive ATA (vs. 33% of SSc-non-ILD patients, *p* = 0.0007), while ACA were significantly less frequent in SSc-ILD patients as opposed to those without ILD (4 vs. 42%, *p* = 0.007). More patients with ILD had received treatment with rituximab (30 vs. 8%, *p* = 0.026), while treatment history with cyclophosphamide and mycophenolate mofetil did not differ significantly between the two groups. History of methotrexate treatment was less frequent in SSc-ILD patients than patients without ILD (13 vs. 25%, *p* = 0.044) (Table 3).

**Table 3:** Demographic, clinical and immunological characteristics of SSc patients with and without ILD.

	**SSc-ILD patients** **(*n* = 23)**	**SSc-non-ILD patients** **(*n* = 12)**	**B-H adjusted *p*-value**
Female gender^*^	21 (91)	11 (92)	0.098
Age^**^	62.3 ± 10.1	58.7 ± 11.1	**0.039**
Smoking (ever)^*^	11 (48)	7 (58)	0.091
Disease duration (years)^#^	5 (0.5–27.3)	12.1 (1–33)	**0.017**
Treatment duration (years)^#^	3 (0–13.5)	1.5 (0–10.3)	0.074
ATA^*^	21 (91)	4 (33)	**0.0007**
ACA^*^	1 (4)	5 (42)	**0.007**
Rituximab (ever)^*^	7 (30)	1 (8)	**0.026**
Cyclophosphamide (ever)^*^	12 (52)	4 (33)	0.056
Mycophenolate mofetil (ever)^*^	14 (61)	5 (42)	0.055
Methotrexate (ever)^*^	3 (13)	3 (25)	**0.044**
Nintedanib (ever)^*^	3 (13)	0	0.066

* Data are expressed as *n* (%).

** Data are expressed as mean ± standard deviation.

# Data are expressed as median (range). B-H, Benjamini-Hochberg.

Bold *p*-values are *p*-values ≤ 0.05.

As expected, SSc-ILD patients presented lower FVC and DLCO (expressed as a percentage of the predicted normal value) than SSc patients without ILD (mean: 83.6 vs. 95.4%, *p* = 0.021 and 53 vs. 82.3%, *p* = 0.004, respectively). Predicted FEF_25−75_ tended to be lower in SSc-ILD patients but did not reach statistical significance (73.8 vs. 79.8%, *p* = 0.061).

Single breath nitrogen washout measurements revealed that SSc-ILD patients had higher values of phase III slope_N2SBW_ (expressed as a percentage of the predicted value) than SSc-non-ILD patients (median: 157 vs. 75%, *p* = 0.038). Then, we used a cutoff value of phase III slope_N2SBW_ predicted ≥120% in order to define small airways dysfunction (16) and we found that both groups included a subset of patients with abnormally increased phase III slope_N2SBW_. However, the frequency of patients with phase III slope_N2SBW_ ≥120% was greater in the SSc-ILD group than in non-ILD (71 vs. 44%, *p* = 0.04). Unexpectedly, patients with ILD had a trend for lower values of predicted CV/VC than SSc-non-ILD patients (median: 34.5 vs. 74%, *p* = 0.052) while the proportion of patients with increased values of CV/VC (defined as ≥120% of the predicted values) (16) did not differ between the two groups (13 vs. 11%, *p* = 0.098). In impulse oscillometry measurements, SSc-ILD patients tended to present increased values of R5–R20 as compared to patients without ILD, but the difference did not reach statistical significance (mean: 0.047 vs. 0.045 kPa/L/s, *p* = 0.094), more likely due to lack of power. Then we used a cutoff value of R5–R20 ≥0.07 kPa/L/s to define small airways dysfunction (11). We found that 50% of SSc-ILD patients and 20% of SSc-non-ILD patients presented R5–R20 ≥0.07 kPa/L/s (*p* = 0.025). X5 and Fres did not differ significantly between the two groups (Table 4).

**Table 4:** Comparison of pulmonary and small airway parameters between SSc-ILD and SSc-non-ILD patients.

	**SSc-ILD patients** **(*n* = 23)**	**SSc-non-ILD patients** **(*n* = 12)**	**B-H adjusted *p*-value**
FVC (% pred)^*^	83.6 ± 26.2	95.4 ± 17.9	**0.021**
FEV1/FVC (%)^*^	82.5 ± 8.1	81.3 ± 5.3	0.078
FEF_25−75_ (% pred)^*^	73.8 ± 22.8	79.8 ± 26.1	0.061
PEF (% pred)^*^	91.9 ± 24.1	85.1 ± 29.9	0.060
sRaw (cmH2O*s)^**^	7.89 (3.4–17.34)	5.18 (1.5–13.78)	**0.020**
Raw (cmH2O*s/L)^**^	2.92 (1.37–21.48)	2.14 (0.53–3.9)	**0.011**
sGaw (1/cmH2O/s)^**^	0.13 (0.06–0.31)	0.20 (0.07–0.72)	**0.022**
Gaw (L/cmH2O/s)^**^	0.37 (0.06–0.73)	0.48 (0.26–1.88)	**0.010**
R_int_ (% pred)^*^	103.4 ± 29.3	95.9 ± 18.0	0.062
CV/VC (% pred)^**^	34.5 (4–193)	74 (8–257)	0.052
CV/VC (% pred) >120%^#^	2 (13)	1 (11)	0.098
phase III slope_N2SBW_ (% pred)^**^	157 (48–585)	75 (15–591)	**0.038**
phase III slope_N2SBW_ (% pred) >120%^#^	10 (71)	4 (44)	**0.040**
DLCO (% pred)^*^	53.0 ± 27.1	82.3 ± 25.6	**0.004**
R4 (kPa/L/s)^*^	0.469 ± 0.155	0.382 ±0.181	**0.030**
R5 (kPa/L/s)^*^	0.370 ± 0.110	0.336 ± 0.115	0.058
R20 (kPa/L/s)^**^	0.297 (0.168–0.616)	0.283 (0.173–0.422)	0.082
R5-R20 (kPa/L/s)^*^	0.047 ± 0.081	0.045 ± 0.066	0.094
R5–R20 ≥0.07 (kPa/L/s)^#^	7 (50)	2 (20)	**0.025**
Fres (Hz)^*^	16.95 ± 653	18.59 ± 5.47	0.069
X6 (kPa/L/s)^*^	−0.156 ± 0.103	−0.179 ± 0.132	0.083

* Data are expressed as mean ± standard deviation.

** Data are expressed as median (range).

# Data are expressed as *n* (%). B-H, Benjamini-Hochberg.

Bold *p*-values are *p*-values ≤ 0.05.

Body plethysmography revealed increased airway resistance in patients with ILD; Raw and sRaw were significantly higher in SSc-ILD patients (median: 2.92 vs. 2.14 cmH_2_O^*^s/L, *p* = 0.011 and 7.89 vs. 5.18 cmH_2_O^*^s, *p* = 0.02, respectively), while sGaw was significantly lower (0.13 vs. 0.2/cmH_2_O/s, *p* = 0.022). Respiratory resistance (% predicted) measured by the interrupter technique (R_int_) tended to be increased in SSc-ILD patients, but the difference did not reach statistical significance (mean: 103.4 vs. 95.9%, *p* = 0.062).

### Comparison of pulmonary and small airways function parameters of SSc-ILD patients between baseline and follow-up visit

Fourteen (*n* = 14) SSc-ILD patients receiving various treatments returned for a second visit, 12 months after the first evaluation and pulmonary and small airways function investigations were repeated. Nine patients received mycophenolate mofetil, two patients received combination of mycophenolate with nintedanib, two were on treatment with rituximab and one had combinational treatment with mycophenolate, rituximab and nintedanib. Number of patients with dyspnea and number of patients with cough did not change significantly between the two visits (71 vs. 71%, *p* = 0.096 and 29 vs. 21%, *p* = 0.092). FVC (% predicted) did not change significantly between baseline and follow-up visit (mean: 86.1 vs. 85.1%, *p* = 0.086), although predicted FEF_25−75_ and peak expiratory flow (PEF) presented a decline across time in SSc-ILD patients (mean: 71.1 vs. 63.5%, *p* = 0.019 and 97.6% vs. 93.1, *p* = 0.045, respectively) and DLCO (% predicted) improved (48.5 vs. 54.9%, *p* = 0.042). Results from both phase III slope_N2SBW_ (expressed as a percentage of the predicted value) and the proportion of SSc-ILD patients with phase III slope_N2SBW_ ≥120% improved between the two visits (mean 290 vs. 177%, *p* = 0.012 and 78 vs. 50%, *p* = 0.034, respectively). However, the ratio CV/VC (% predicted) deteriorated between the two visits (42.5–77%, *p* = 0.021). In IOS measurements, R5–R20 tended to improve from the first to the second visit (0.053 vs. 0.04 kPa/L/s, *p* = 0.068). Nevertheless, the frequency of SSc-ILD patients with R5–R20 ≥0.07 kPa/L/s decreased in the follow-up visit (57 vs. 33%, *p* = 0.035) while X5 and Fres did not change significantly between the two visits (Table 5). Raw as measured by body plethysmography presented an improvement (mean: 3.05 vs. 2.69 cmH_2_O^*^s/L, *p* = 0.044), but sRaw and sGaw did not change significantly. R_int_ also remained unchanged between the two visits.

**Table 5:** Comparison of pulmonary and small airway parameters of 14 SSc-ILD patients between baseline and follow-up visit.

	**Baseline visit** **(*n* = 14)**	**Follow-up visit (*n* = 14)**	**B-H adjusted *p*-value**
Dyspnea^#^	10 (71)	10 (71)	0.096
Cough^#^	4 (29)	3 (21)	0.092
FVC (% pred)^*^	86.1 ± 25.5	85.1 ± 23.8	0.086
FEV1/FVC (%)^*^	81.5 ± 7.5	80.0 ± 5.5	0.052
FEF_25−75_ (% pred)^*^	71.1 ± 19.3	63.5 ± 13.4	**0.019**
PEF (% pred)^*^	97.6 ± 17.0	93.1 ± 17.1	**0.045**
sRaw (cmH2O*s)^**^	5.59 (3.4–9.96)	6.08 (3.38–18.08)	0.075
Raw (cmH2O*s/L)^*^	3.05 ± 0.98	2.69 ±1.42	**0.044**
sGaw (1/cmH2O/s)^**^	0.18 (0.1–0.31)	0.17 (0.06–0.49)	0.089
Gaw (L/cmH2O/s)^**^	0.37 (0.2–0.6)	0.4 (0.17–1.88)	**0.043**
R_int_ (% pred)^*^	110.1 ± 27.7	112.7 ± 26.7	0.077
CV/VC (% pred)^**^	42.5 (20–193)	77 (20–170)	**0.021**
CV/VC (% pred) >120%^#^	1 (10)	2 (17)	0.097
phase III slope_N2SBW_ (% pred)^*^	290 ± 176	177 ± 142	**0.012**
phase III slope_N2SBW_ (% pred) >120%^#^	7 (78)	6 (50)	**0.034**
DLCO corr (% pred)^*^	48.5 ± 19.6	54.9 ± 19.5	**0.042**
R4 (kPa/L/s)^*^	0.531 ± 0.138	0.439 ± 0.143	**0.014**
R5 (kPa/L/s)^*^	0.436 ± 0.089	0.351 ± 0.078	**0.003**
R20 (kPa/L/s)^**^	0.326 (0.217–0.616)	0.304 (0.195–0.43)	**0.036**
R5–R20 (kPa/L/s)^*^	0.053 ± 0.083	0.040 ± 0.071	0.068
R5–R20 ≥0.07 (kPa/L/s)^#^	4 (57)	4 (33)	**0.035**
Fres (Hz)^*^	18.50 ± 6.88	17.89 ± 5.89	0.082
X6 (kPa/L/s)^**^	−0.118 (−0.305 to −0.098)	−0.155 (−0.394 to −0.104)	0.051

* Data are expressed as mean ± standard deviation.

** Data are expressed as median (range).

# Data are expressed as *n* (%). B-H, Benjamini-Hochberg.

Bold *p*-values are *p*-values ≤ 0.05.

## Discussion

This prospective study investigated the pulmonary and small airways function among SSc patients with and without radiological evidence of ILD and explored the potential effect of treatment upon small airways by using a vast array of pulmonary function tests. To our knowledge, this is the first study in SSc exploring the diagnostic yield of 3 distinct methodologies to detect small airways dysfunction. Among R5–R20, phase III slope_N2SBW_ and CV/VC, phase III slope_N2SBW_ showed the higher diagnostic performance, detecting small airway dysfunction in 60% of SSc patients. It should be stressed that phase III slope_N2SBW_ was elevated in nearly half (4 out of 9) of SSc patients with no evidence of ILD in HRCT scan which suggests that it could be used for the early detection of small airways dysfunction in these patients. Most phase III slope_N2SBW_ positive patients had ILD findings in HRCT, connoting an association between ILD and small airways dysfunction. The fact that HCRT findings for small airway disease were comparable between SSc patients with normal and elevated phase III slope_N2SBW_ values, suggests that HRCT is not sensitive enough for detecting small airway dysfunction.

Using HRCT to define ILD, as it is commonly happening in clinical practice unraveled that only 22% of SSc-ILD patients had radiographic features of SAD whereas SSc-non-ILD patients had no such findings, providing further proof that HRCT is insensitive for the detection of small airway dysfuction in SSc patient. Disease duration was significantly shorter in SSc-ILD patients compared to those without ILD, implying that SSc itself may in some cases involve the small airways during long disease course, since non ILD patients also developed SAD at some extent. Taken together, the previously mentioned contemporary tests/parameters in our study imply that ILD of SSc is associated with small airways dysfunction.

Assessment of airway resistance by body plethysmography revealed increased Raw, sRaw and decreased sGaw, in patients with ILD compared to non-ILD patients. Increased Raw and sRaw reflect central and peripheral airways obstruction, whereas decreased sGaw seems to be a more sensitive measure of small airways dysfunction (25, 32). Gender and smoking history did not differ between the two groups. However, SSc-ILD patients were relatively older than patients without ILD (mean: 62.3 vs. 58.7 years old, *p* = 0.039), though physiologically this age difference is of minimal significance. It should be noted though that older age at disease onset is a risk factor for development of ILD in SSc patients (33) and this may partly explain the age difference between the two groups of our study.

Small airways function in patients with ILD has been partly addressed by a number of studies. Studies using conventional PFTs (FEF_25−75_ and maximal expiratory flow at 25% of the FVC, MEF_25_) have shown conflicting results (12, 13, 34, 35) due mostly to the inherent variability of these measurements, among other reasons. Mikamo et al. studied patients with ILD, either idiopathic or secondary to systemic autoimmune rheumatic diseases including SSc, and found radiographic features of SAD on HRCT, normal conventional PFTs and abnormal values of specific PFTs indices, such as R5–R20, X5 and Fres, implying small airways dysfunction. However, the authors did not make distinction between patients with idiopathic or autoimmune ILD (20) and used only a IOS for small airway dysfunction detection. Similarly, Aronsson et al., by employing the IOS technique, compared small airways function between 78 SSc patients and 26 healthy controls and found significantly increased R5–R20 and Fres in the SSc patients, suggesting small airways dysfunction (10). Bonifazi et al. utilized both IOS and HRCT in 93 SSc patients and demonstrated that an increased number of SSc patients had small airways dysfunction, defined as R5–R20 ≥0.07 kPa/L/s compared to healthy controls, and that 24.7% of SSc patients had at least one radiographic features of SAD on HRCT (11). However, in these studies, no distinction or comparison was made between ILD and non-ILD SSc patients. Silva et al. investigated small airways function in SSc patients, independently of ILD, by means of the N_2_SBW technique and found impaired small airways function, as attested by abnormally increased predicted values of phase III slope_N2SBW_ >120% or CV/VC (% predicted) >120% (16).

To the extent of our knowledge, no study has applied the vast array of tests that we did in our study, which allows us to make inferences about the diagnostic performance of the tests and is a major strength of our study.

Interestingly, very few studies have highlighted the underlying pathogenetic mechanisms of SAD in association with ILD of either idiopathic or autoimmune etiology. The proposed mechanisms include either expansion of the inflammation from lung interstitium to the bronchioles or a direct insult of the main underlying disease into small airways (17, 19, 21). Although ILD has been classically considered a diffuse parenchymal disorder, a number of histopathologic studies have demonstrated small airways involvement in non-autoimmune ILD (19, 21). Reduction of small airways diameter and a variety of histopathologic findings such as peribronchiolar inflammation, fibrosis, epithelial metaplasia and bronchus-associated lymphoid tissue (BALT) have been observed in lung biopsy of patients with idiopathic pulmonary fibrosis (IPF) and non-autoimmune NSIP, UIP, even in areas without extensive fibrosis, suggesting that small airways may be involved primarily in ILD (17, 36). The fact that SSc-non-ILDs may also develop small airways dysfunction suggests that both mechanisms may be implicated in SSc and that contemporary pulmonary function tests may detect early stages of small airways disease before the development of structural lesions on HRCT scan.

In our study the effect of treatment on small airways function among SSc-ILD patients was controversial. Under various treatment modalities and after 1 year of follow-up, changes in pulmonary tests followed different patterns: some were found unchanged (sRaw, sGaw and R_int_), while others showed an improvement of small airways function (Raw, % predicted and frequency of >120% of phase III slope_N2SBW_, and R5–R20 >0.07 kPa/L/s). Such conflicting findings may reflect treatment heterogeneity and timing during disease course as well as the different extent and contribution of ILD inflammation on small airway dysfunction among SSc-ILD patients. However, it seems that a subset of patients may benefit from early therapeutic interventions of SSc-associated ILD with the potential to prevent also small airways dysfunction by controlling efficiently the inflammatory component of lung interstitium. To this end, future studies with large and well-characterized cohorts may reveal criteria to identify those SSc-ILD patients who may respond to treatment in terms of SAD and propose the proper regimen and timing during the disease course.

The present study has several limitations. Firstly, the sample of patients is rather small to reveal statistically significant associations among some variables or to perform multivariate analysis for potential confounders. Secondly, the study did not include healthy or disease controls to make useful comparisons. Thirdly, there is no gold standard defining sensitivity and specificity of N_2_SBW and IOS evaluating small airways dysfunction. Finally, lung biopsy was not performed to document small airways inflammation and damage or to elucidate pathogenetic mechanisms.

In conclusion, our study by utilizing a vast array of pulmonary function tests and especially phase III slope_N2SBW_, and R5–R20, showed that small airways dysfunction may be associated with SSc-related ILD. A smaller subset of SSc-ILD patients display radiographic features of small airways disease on HRCT, suggesting that phase III slope_N2SBW_, especially when combined with R5–R20 may be able to detect small airways abnormalities before structural damage evident on imaging. In addition, ILD therapy may improve small airways function in a subset of patients. Taken together, it seems that small airways dysfunction is part of the spectrum of lung involvement in SSc and can be early diagnosed with specific pulmonary function tests before the development of radiographically evident structural damage. The fact that ILD treatment may also affect small airways function in some patients, raises the clinical question of early therapeutic intervention in these SSc-ILD patients. Studies with larger number of SSc patients are required to confirm this observation and to clarify the effect of immunomodulatory treatment on small airways function of these patients, while histopathologic studies are anticipated to contribute to the investigation of the pathogenetic mechanisms mediating these processes.

## Data availability statement

The raw data supporting the conclusions of this article will be made available by the authors, without undue reservation.

## Ethics statement

The studies involving human participants were reviewed and approved by Ethics Committee of the School of Medicine, National and Kapodistrian University of Athens. The patients/participants provided their written informed consent to participate in this study.

## Author contributions

PP, AG, PV, TV, and AT made substantial contributions to the conception, design of the study, the analysis, interpretation of data, and drafted the manuscript. PV, TV, and AT were responsible for supervision. PP, AG, VG, AK, and EC made substantial contributions to data collection and interpretation. VP, KM, and DF made substantial contributions to data analysis and interpretation. All authors contributed to the article and approved the submitted version.

## Funding

The current work has been funded by the Research Institute of Systemic Autoimmune Diseases.

## Conflict of interest

The authors declare that the research was conducted in the absence of any commercial or financial relationships that could be construed as a potential conflict of interest.

## Publisher's note

All claims expressed in this article are solely those of the authors and do not necessarily represent those of their affiliated organizations, or those of the publisher, the editors and the reviewers. Any product that may be evaluated in this article, or claim that may be made by its manufacturer, is not guaranteed or endorsed by the publisher.
