# Supplementary material for: Small airways dysfunction in patients with systemic sclerosis and interstitial lung disease
Source: Front Med (Lausanne). 2022 Nov 14;9:1016898. doi: 10.3389/fmed.2022.1016898 (PMC9702077; doi:10.3389/fmed.2022.1016898)
Supplement: Supplementary file 1 [file Data_Sheet_1.docx]

**Supplementary material**

**1 Patients and Methods**

**1.1 Assessment of pulmonary and small airways function**

**1.1.1 Spirometry and measurement of diffusing capacity**

Classic spirometry was performed with the Q-Box (Cosmed Micro Quark, Italy). Age, gender, height and weight were recorded and predicted values of respiratory parameters were calculated automatically, by comparing each respiratory parameter of patients to an average for a person of the same gender, height and age. DLCO was measured using the single breath holding technique with CH_4_ and CO as tracer gases. DLCO was corrected according to the hemoglobin concentration. The predicted values of the European Respiratory Society were used ([32](#_ENREF_32), [33](#_ENREF_33)).

**1.1.2 Body plethysmography technique**

Static lung volumes [total lung capacity (TLC), residual volume (RV)] and airway resistance and conductance (Raw, Gaw) were measured using the body plethysmography technique. The assessment of pulmonary function by body plethysmography begins with breathing at rest, followed by the shutter maneuver during which the airflow is deliberately blocked by transient occlusion. After opening of the shutter, an expiratory reserve volume (ERV) effort and an inspiratory vital capacity effort (IVC) were performed allowing the computation of RV and TLC. During assessment the box is closed with an airtight seal, except for a small controlled leak which is used in order to stabilize the internal pressure. One pressure transducer measures the pressure inside the Q-box relative to ambient pressure, while another one is placed close to the mouth for monitoring mouth pressure during the shutter maneuver. The predicted values of the European Respiratory Society were used ([33](#_ENREF_33)).

**1.1.3 Single breath nitrogen washout technique**

The single breath nitrogen washout technique was performed with a mouthpiece connected to Q-Box, by inhaling 100% oxygen from RV to TLC followed by a slow vital capacity (SVC) exhalation. Change in the concentration of nitrogen between 25% and 75% of the exhaled volume (phase III slope_N2SBW_) and the closing volume/vital capacity ratio (CV/VC) were estimated and reported as percentage of the predicted values. The predicted values of the European Respiratory Society were used ([34](#_ENREF_34)). We used values of phase III slope_N2SBW_ (% predicted) ≥ 120% and CV/VC (% predicted) ≥ 120% to define small airways dysfunction ([11](#_ENREF_11)).

**1.1.4 Interrupter technique**

Respiratory resistance was measured by the interrupter technique (R_int_) and predicted values were those of the Asthma UK Initiative ([22](#_ENREF_22)). The interrupter technique is performed using a flowmeter, a pressure measurement device, and a flow interruption system (valve). Interruptions were triggered at peak tidal flow. Mouth pressure was calculated using the two-point linear back extrapolation to 0 ms after the onset of interruption. The minimum number of technically acceptable tracings required were six.

**1.1.5 Impulse oscillometry (IOS)**

Measurement of the respiratory resistance and reactance was performed with impulse oscillometry equipment (MS-IOS Jaeger) according to ERS protocols ([35](#_ENREF_35)). IOS was carried out before spirometry because forced expiration might affect airway tone and predicted values of the European Respiratory Society were used ([36](#_ENREF_36)). Pressure oscillations generated by a loudspeaker were superimposed onto normal tidal breathing through a mouthpiece for 30 to 45 s, which ranged from 4 to 35 Hz in frequency. Sitting upright, patients were asked to place the noseclip and exert manual compression on their faces to minimize the influence of cheek vibration and air leak. Three trials were conducted and mean values of the following parameters were recorded: respiratory resistance at 6 Hz (R5) and 20 Hz (R20), R5-R20, X6 and Fres. We defined small airways dysfunction as R5-R20 ≥ 0.07 kPa/L/s ([6](#_ENREF_6)).

**References**

1. Cotes JE, Chinn DJ, Quanjer PH, Roca J, Yernault JC. Standardization of the Measurement of Transfer Factor (Diffusing Capacity). *The European respiratory journal* (1993) 6 Suppl 16:41-52. Epub 1993/03/01. doi: 10.1183/09041950.041s1693.

2. Quanjer PH, Tammeling GJ, Cotes JE, Pedersen OF, Peslin R, Yernault JC. Lung Volumes and Forced Ventilatory Flows. Report Working Party Standardization of Lung Function Tests, European Community for Steel and Coal. Official Statement of the European Respiratory Society. *The European respiratory journal Supplement* (1993) 16:5-40. Epub 1993/03/01.

3. Robinson PD, Latzin P, Verbanck S, Hall GL, Horsley A, Gappa M, et al. Consensus Statement for Inert Gas Washout Measurement Using Multiple- and Single- Breath Tests. *The European respiratory journal* (2013) 41(3):507-22. Epub 2013/02/12. doi: 10.1183/09031936.00069712.

4. Silva BRA, Rufino R, Costa CH, Vilela VS, Levy RA, Lopes AJ. Ventilation Distribution and Small Airway Function in Patients with Systemic Sclerosis. *Revista portuguesa de pneumologia* (2017) 23(3):132-8. Epub 2017/03/05. doi: 10.1016/j.rppnen.2017.01.004.

5. Merkus PJ, Stocks J, Beydon N, Lombardi E, Jones M, McKenzie SA, et al. Reference Ranges for Interrupter Resistance Technique: The Asthma Uk Initiative. *The European respiratory journal* (2010) 36(1):157-63. Epub 2009/12/25. doi: 10.1183/09031936.00125009.

6. King GG, Bates J, Berger KI, Calverley P, de Melo PL, Dellacà RL, et al. Technical Standards for Respiratory Oscillometry. *European Respiratory Journal* (2020) 55(2):1900753. doi: 10.1183/13993003.00753-2019.

7. Oostveen E, MacLeod D, Lorino H, Farré R, Hantos Z, Desager K, et al. The Forced Oscillation Technique in Clinical Practice: Methodology, Recommendations and Future Developments. *The European respiratory journal* (2003) 22(6):1026-41. Epub 2003/12/19. doi: 10.1183/09031936.03.00089403.

8. Bonifazi M, Sverzellati N, Negri E, Pomponio G, Seletti V, Bonini M, et al. Increased Prevalence of Small Airways Dysfunction in Patients with Systemic Sclerosis as Determined by Impulse Oscillometry. *Rheumatology (Oxford, England)* (2020) 59(3):641-9. Epub 2019/08/23. doi: 10.1093/rheumatology/kez340.

Supplementary Table 1. Characteristics of SSc patients’ cohort.

|  | **SSc patients (n=35)** |
| --- | --- |
| Female gender* | 32 (91) |
| Age** | 61.1 ± 10.7 |
| Smoking (ever)* | 18 (51) |
| Disease duration (years) ^#^ | 6.3 (0.5-33) |
| Treatment duration (years) ^#^ | 2.5 (0-13.5) |
| Diffuse cutaneous involvement* | 12 (34) |
| Dyspnea* | 21 (60) |
| Cough* | 4 (11) |
| ATA* | 25 (71) |
| ACA* | 6 (17) |
| Rituximab (ever)* | 8 (23) |
| Cyclophosphamide (ever)* | 16 (46) |
| Mycophenolate mofetil (ever)* | 19 (54) |
| Methotrexate (ever)* | 6 (17) |
| Nintedanib (ever)* | 3 (9) |

Abbreviations: ATA = anti-topoisomerase I antibodies, ACA = anti-centromere antibodies. *Data are expressed as n(%). **Data are expressed as mean ± standard deviation. ^#^Data are expressed as median (range)

Supplementary Table 2. Comparison of SSc patients characteristics with and without increased R5-R20.

|  | **SSc patients with R5-R20 ≥ 0.07 kPa/L/s (n=9)** | **SSc patients with R5-R20 < 0.07 kPa/L/s (n=15)** | **B-H adjusted**  **p-value** |
| --- | --- | --- | --- |
| Female gender* | 8 (89) | 13 (87) | 0.081 |
| Age** | 60.6 ± 11.7 | 60.9 ± 11.3 | 0.075 |
| Smoking (ever)* | 4 (44) | 8 (53) | 0.087 |
| **Disease duration (years) ^#^** | **5 (0.5-15.5)** | **9.2 (1-33)** | **0.048** |
| Treatment duration (years) ^#^ | 2 (0-5.5) | 1.5 (0-10) | 0.072 |
| **ILD*** | **7 (78)** | **7 (47)** | **0.030** |
| ATA* | 6 (67) | 11 (73) | 0.080 |
| **ACA*** | **3 (33)** | **2 (13)** | **0.036** |
| **Rituximab (ever)*** | **3 (33)** | **1 (7)** | **0.022** |
| Cyclophosphamide (ever)* | 3 (33) | 6 (40) | 0.082 |
| Mycophenolate mofetil (ever)* | 4 (44) | 8 (53) | 0.092 |
| Methotrexate (ever)* | 1 (11) | 3 (20) | 0.083 |
| Nintedanib (ever)* | 2 (22) | 0 (0) | 0.025 |
| FVC (%pred)** | 98.7 ± 16.4 | 94.5 ± 19.5 | 0.054 |
| **FEV1/FVC (%)**** | **76.7 ± 5.8** | **83 ± 4.8** | **0.010** |
| **FEF_25-75_ (%pred)**** | **62.2 ± 19.9** | **88.9 ± 24.4** | **0.011** |
| PEF (% pred)** | 87.8 ± 17.4 | 91.6 ± 33.3 | 0.065 |
| **sRaw (cmH2O*s)**** | **8.36 ± 2.33** | **6.59 ± 3.78** | **0.031** |
| **Raw (cmH2O*s/L)^#^** | **3.3 (1.97-4.97)** | **2.3 (0.53-4.12)** | **0.015** |
| **sGaw (1/cmH2O/s)^#^** | **0.12 (0.08-0.23)** | **0.19 (0.06-0.72)** | **0.028** |
| **Gaw (L/cmH2O/s)^#^** | **0.31 (0.2-0.51)** | **0.44 (0.06-1.88)** | **0.017** |
| **R_int_ (%pred)^#^** | **125.5 (81-164)** | **85 (53-128)** | **0.019** |
| **CV/VC (% pred)^#^** | **22 (4-132)** | **34.5 (11-257)** | **0.042** |
| CV/VC (% pred) > 120% * | 1 (13) | 1 (8) | 0.099 |
| phase III slope_N2SBW_ (% pred)^#^ | 120 (63-497) | 126 (15-591) | 0.074 |
| phase III slope_N2SBW_ (% pred) > 120% * | 4 (57) | 7 (58) | 0.079 |
| **DLCO (% pred) **** | **68.9 ± 26.7** | **77.5 ± 25.6** | **0.046** |
| **R4 (kPa/L/s)**** | **0.585 ± 0.135** | **0.342 ±0.120** | **0.003** |
| **R5 (kPa/L/s)**** | **0.424 ± 0.074** | **0.315 ± 0.113** | **0.012** |
| R20 (kPa/L/s)^#^ | 0.295 (0.229-0.371) | 0.287 (0.173-0.616) | 0.062 |
| **R5-R20 (kPa/L/s)**** | **0.123 ± 0.044** | **-0.001 ± 0.047** | **0.002** |
| **Fres (Hz)**** | **21.79 ± 3.76** | **15.13 ± 5.97** | **0.007** |
| **X6 (kPa/L/s)**** | **-0.279± 0.094** | **-0.094 ± 0.050** | **0.004** |
| **Dyspnea*** | **3 (33)** | **8 (53)** | **0.044** |
| **Cough*** | **2 (22)** | **0** | **0.026** |
| Presence of SAD findings on HRCT* | 1 (7) | 1 (12) | 0.091 |

*Data are expressed as n(%). **Data are expressed as mean ± standard deviation. ^#^Data are expressed as median (range), B-H: Benjamini-Hochberg

Supplementary Table 3. Comparison of SSc patients characteristics with and without increased phase III slope_N2SBW_.

|  | **SSc patients with phase III slope_N2SBW_ (% pred) ≥ 120% (n=14)** | **SSc patients with phase III slope_N2SBW_ (% pred) < 120% (n=9)** | **B-H adjusted**  **p-value** |
| --- | --- | --- | --- |
| Female gender* | 13 (93) | 8 (89) | 0.097 |
| **Age^#^** | **62 (32-71)** | **61 (43-69)** | **0.045** |
| **Smoking (ever)*** | **10 (71)** | **6 (67)** | **0.016** |
| Disease duration (years) ^#^ | 5 (1-33) | 3 (1-15.5) | 0.073 |
| **Treatment duration (years)** | **2.8 (0-10)** | **0.8 (0-8)** | **0.012** |
| **ILD*** | **10 (71)** | **4 (44)** | **0.042** |
| ATA* | 10 (71) | 6(67) | 0.078 |
| ACA* | 2 (14) | 2 (22) | 0.082 |
| Rituximab (ever)* | 3 (21) | 1 (11) | 0.080 |
| Cyclophosphamide (ever)* | 7 (50) | 3 (33 | 0.065 |
| Mycophenolate mofetil (ever)* | 7 (50) | 5 (56) | 0.090 |
| Methotrexate (ever)* | 2 (14) | 1 (11) | 0.075 |
| Nintedanib (ever)* | 1 (7) | 0 (0) | 0.099 |
| **FVC (%pred)**** | **91.9 ± 16.6** | **106.3 ± 11.7** | **0.008** |
| **FEV1/FVC (%)**** | **80.2 ± 5.1** | **82.8 ± 4.4** | **0.035** |
| **FEF_25-75_ (%pred)**** | **74.3 ± 26.7** | **91.7 ± 14.9** | **0.018** |
| **PEF (% pred)**** | **103.9 ± 16** | **88.7 ± 26.9** | **0.021** |
| sRaw (cmH2O*s)^#^ | 5.86 (3.4 -9.84 ) | 5.4 (1.5-9.71) | 0.070 |
| **Raw (cmH2O*s/L)^#^** | **2.7 (1.83-4.97)** | **2.3 (0.55-4.4)** | **0.034** |
| **sGaw (1/cmH2O/s)^#^** | **0.18 (0.06-0.31)** | **0.19 (0.1-0.72)** | **0.055** |
| **Gaw (L/cmH2O/s)^#^** | **0.38 (0.06-0.57)** | **0.44 (0.23-1.88)** | **0.037** |
| **R_int_ (%pred)**** | **93.9 ± 25.3** | **112.2 ± 30** | **0.031** |
| **CV/VC (% pred)^#^** | **55 (6-193)** | **24 (4-257)** | **0.024** |
| CV/VC (% pred) > 120% * | 1 (7) | 1 (11) | 0.084 |
| **phase III slope_N2SBW_ (% pred)^#^** | **356 (120-591)** | **63 (15-118)** | **0.001** |
| **DLCO (% pred)**** | **62.1 ± 20** | **90.3 ± 23.9** | **0.003** |
| **R4 (kPa/L/s)**** | **0.471 ± 0.179** | **0.406 ± 0.131** | **0.046** |
| **R5 (kPa/L/s)**** | **0.392 ± 0.120** | **0.330 ± 0.092** | **0.036** |
| **R20 (kPa/L/s)**** | **0.352 ± 0.131** | **0.280 ± 0.045** | **0.028** |
| R5-R20 (kPa/L/s)** | 0.040 ± 0.090 | 0.050± 0.074 | 0.070 |
| R5-R20 ≥ 0.07 (kPa/L/s) * | 4 (29) | 3 (33) | 0.096 |
| Fres (Hz)** | 16.86 ± 5.59 | 17.54 ± 6.7 | 0.072 |
| X6 (kPa/L/s)** | -0.177 ± 0.120 | -0.213 ± 0.087 | 0.063 |
| **Dyspnea*** | **10 (71)** | **3 (33)** | **0.017** |
| **Cough*** | **0** | **1 (11)** | **0.043** |
| Presence of SAD findings on HRCT* | 2 (14) | 1 (12) | 0.093 |

*Data are expressed as n(%). **Data are expressed as mean ± standard deviation. ^#^Data are expressed as median (range), B-H: Benjamini-Hochberg
